# Combustion Synthesis of SrAl_2_O_4_: Eu^2+^, Dy^3+^ Phosphorescent Pigments for Glow-in-the-Dark Safety Markings

**DOI:** 10.3390/nano13040687

**Published:** 2023-02-10

**Authors:** Radu Lazău, Robert Ianoș, Cornelia Păcurariu, Diana Aylin Căpraru, Andrei Racu, Vlad Cornea

**Affiliations:** 1 Faculty of Industrial Chemistry and Environmental Engineering, Politehnica University of Timișoara, P-ța Victoriei No. 2, 300006 Timișoara, Romania; 2 National Institute of Research & Development for Electrochemistry and Condensed Matter, INCEMC, Str. Dr. A. Păunescu Podeanu No. 144, 300569 Timisoara, Romania; 3 AZUR S.A., Constructorilor Blvd. No. 1-3, 300571 Timișoara, Romania

**Keywords:** phosphorescent pigments, glow-in-the-dark, safety markings, combustion synthesis, perchlorates

## Abstract

This study deals with SrAl_2_O_4_: Eu^2+^, Dy^3+^ phosphor pigments prepared by an optimized perchlorate-assisted combustion synthesis and tested for developing glow-in-the-dark safety markings. Recipes with different oxidizer/fuel ratios were designed to create an in-situ reducing-reaction atmosphere and promote Eu^3+^ → Eu^2+^ reduction, which is responsible for the specific long-lasting, green emission of the pigments. The obtained data proved the efficiency of glycine-rich mixtures (up to 200% glycine excess), which led to improved optical features, as compared to the reference stoichiometric sample. The best results in terms of emission intensity and decay time were obtained in the case of 100% glycine excess. The sample with optimum emission characteristics was successfully tested in making glow-in-the-dark coatings applied to two different substrates and using pigment concentrations between 10 and 33% weight.

## 1. Introduction

Europium- and dysprosium-doped strontium aluminate (SrAl_2_O_4_: Eu^2+^, Dy^3+^) is, alongside other luminescent materials [1,2,3,4,5,6], one of the most important phosphors in current use [7,8]. Its intense green emission and long afterglow make SrAl_2_O_4_: Eu^2+^, Dy^3+^ an extremely interesting material for a broad range of applications: emergency and safety lighting [9], roadway markings [10,11], anti-counterfeiting labels [12], phosphor-coated bricks [13], and different composite materials [14,15,16,17,18].

The long-lasting emission of strontium aluminate doped with europium and dysprosium relies on Eu^2+^ dopant ions, which act as emission centers, while Dy^3+^ co-doping ions generate traps responsible for long afterglow. Different emission mechanisms were proposed and discussed in the literature in the last two decades, and different experimental techniques were used to provide confirmations and undersign the contribution of europium and its oxidation state [19]. It is generally admitted that the optical properties of europium- and dysprosium-doped strontium aluminate are highly related to several factors, amongst which the europium-oxidation state (namely, Eu^2+^) plays a key role.

Therefore, despite the different preparation routes (ceramic, sol-gel, citrate, etc.), the annealing process is conducted at high temperature (above 1200 °C) to promote the formation of the SrAl_2_O_4_ or other crystalline host structure while ensuring a reducing atmosphere (H_2_, active carbon, CO) to facilitate the stabilization of Eu^2+^ instead of Eu^3+^ [4,5,6,9,19,20].

Due to the remarkable advantages, combustion synthesis is frequently used for the preparation of SrAl_2_O_4_: Eu^2+^, Dy^3+^ phosphors [21,22] and other SrAl_2_O_4_-related materials [23,24]. There are (at least) three main reasons why combustion synthesis is recommended for the preparation of SrAl_2_O_4_: Eu^2+^, Dy^3+^ phosphor. The first one is related to the remarkable potential of obtaining crystalline SrAl_2_O_4_: Eu^2+^, Dy^3+^ without the need for a time-consuming and costly annealing stage. Unlike other synthesis procedures, the combustion route relies on the heat released during the highly exothermic redox process. This means that the amount of energy required for solid-state reactions to occur is provided by the chemical reaction itself, not by external heating equipment such as an electric furnace. The second reason for using the combustion method deals with the possibility of generating in situ a reducing atmosphere during the preparation process, which facilitates the reduction of Eu^3+^ to Eu^2+^ and enhances the overall optical properties. The third reason has to do with the concentration of crystal-lattice defects, which plays a significant role in room-temperature persistent luminescence [19,25,26,27]. Very high heating and cooling rates are typical features of combustion synthesis, which often lead to an increased concentration of lattice defects.

In this study, SrAl_2_O_4_: Eu^2+^, Dy^3+^ phosphors were prepared by an optimized perchlorate-assisted combustion synthesis and tested for developing glow-in-the-dark safety markings.

## 2. Materials and Methods

SrAl_2_O_4_: Eu^2+^, Dy^3+^ samples were prepared by solution-combustion synthesis, starting from metal perchlorates and mixtures of urea (CH_4_N_2_O–Merck, Darmstadt, Germany, 98%) and glycine (C_2_H_5_NO_2_–Fluka, Buchs, Switzerland, 99%). Metal perchlorates were prepared by dissolving appropriate amounts of SrCO_3_ (Sigma-Aldrich, Steinheim am Albuch, Germany, 99.9%) and Al(OH)_3_ (Alfa Aesar, Kandel, Germany, 92% Al_2_O_3_ content) in HClO_4_ (Honeywell, Seelze, Germany, 70%, density 1.68 g/cm^3^) under magnetic stirring (300 rpm) and heating to boiling point. Eu(NO_3_)_3_·6H_2_O (Acros Organics, Geel, Belgium, 99.9%) and Dy(NO_3_)_3_·5H_2_O (Alfa Aesar, Kandel, Germany, 99.99%) were used for doping the SrAl_2_O_4_. Recipes were designed for obtaining 0.04 moles of Sr_0.97_Eu_0.01_Dy_0.02_Al_2_O_4_. In order to maximize the luminescent properties, 2 molar% B_2_O_3_ was added in all samples in the form of H_3_BO_3_ (Merck, Darmstadt, Germany, 99.8%).

Samples with different oxidizer/fuel ratios were designed, namely, stoichiometric ratio (sample A) and glycine fuel-rich samples: D (25% glycine excess), E (50% glycine excess), F (100% glycine excess), and G (200% glycine excess). The stoichiometric sample (A) refers to the Sr_0.97_Eu_0.01_Dy_0.02_Al_2_O_4_ composition, which was prepared starting from a mixture with the following molar composition: strontium perchlorate/europium (III) nitrate/dysprosium nitrate/glycine/aluminum perchlorate/urea = 0.97/0.01/0.02/1.128/2/8. In the case of samples with fuel-rich composition, an excess of glycine was used with the purpose of creating a reducing atmosphere so that the molar coefficient of glycine became 1.410 (sample D-25% glycine excess), 1.692 (sample E-50% glycine excess), 2.256 (sample F-100% glycine excess), and 3.384 (sample G-200% glycine excess).

The porcelain evaporating dish containing the homogeneous, clear solution of strontium and aluminum perchlorates, europium and dysprosium nitrates, urea, glycine, and boric acid was inserted into the electric furnace preheated to 500 °C. Combustion reactions were accompanied by a bright-white incandescence and the evolution of a large volume of colorless gases. The resulting solid material was ground by hand using a mortar and pestle and further characterized.

The heating behavior of the precursor mixtures was investigated by thermal analysis up to 700 °C using a Netzsch STA 449C instrument (NETZSCH-Gerätebau GmbH, Selb, Germany) and alumina crucibles. The TG-DSC curves were recorded under an air atmosphere. The temperature evolution during combustion reactions was monitored by thermal imaging using a FLIR T 640 infrared camera (Teledyne Flir, Wilsonville, OR, USA); in this case, the reactions were ignited on a heating mantle. The phase composition of the samples was assessed by X-ray diffraction using a Rigaku Ultima IV (Rigaku Corporation, Tokyo, Japan), Cu_Kα_ radiation. The specific surface area of the samples was measured by the BET (Brunauer, Emmett, Teller) method using a Micromeritcs ASAP 2020 (Micromeritics Instrument Corp., Norcross, GA, USA) instrument and nitrogen as an adsorption gas at liquid-nitrogen temperature after degassing the samples at 400 °C for 5 h under 6 µmHg vacuum.

Particle morphology was investigated using an FEI Inspect S scanning electron microscope coupled with energy-dispersive X-ray analysis (EDX) (FEI Company, Hillsboro, OR, USA). The photoluminescence properties of the samples were visually assessed under the UV illumination at 365 nm using a VL-215.LC, 30 W lamp (Vilber, Marne-la-Vallée, France), and white-light (4000 K) illumination with a 100 W, 8000 lm LED lamp (Ecoplanet s.r.o., Bilina, Czech Republic). The room-temperature luminescence measurements were performed with an FLS-980 Edinburgh Instruments (Edinburgh Instruments, Livingston, United Kingdom) photoluminescence spectrometer. For stationary and time-resolved measurements the excitation source used was the Xe lamp (450 W) and the detector used was a PMT Hamamatsu R928P (Hamamatsu Photonics K.K, Electron Tube Division, Iwata, Japan).

## 3. Results

**Figure 1:**
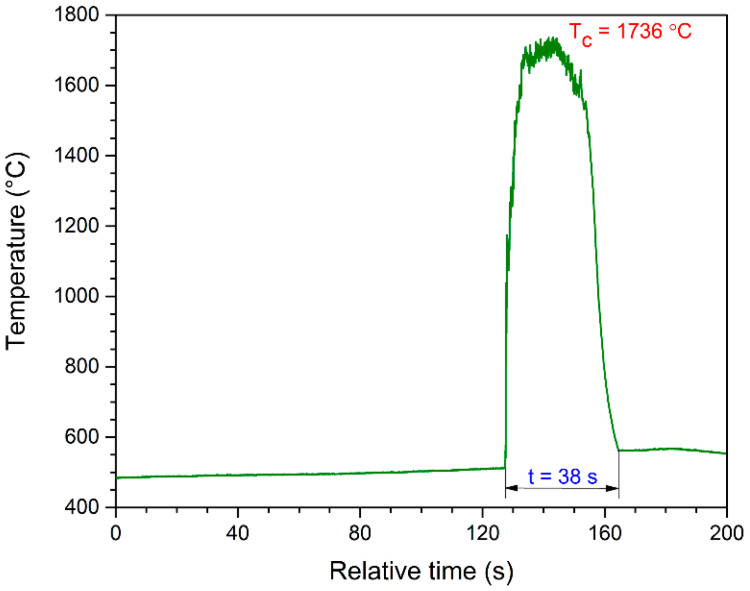
The temperature evolution during the combustion synthesis of sample F.

**Figure 2:**
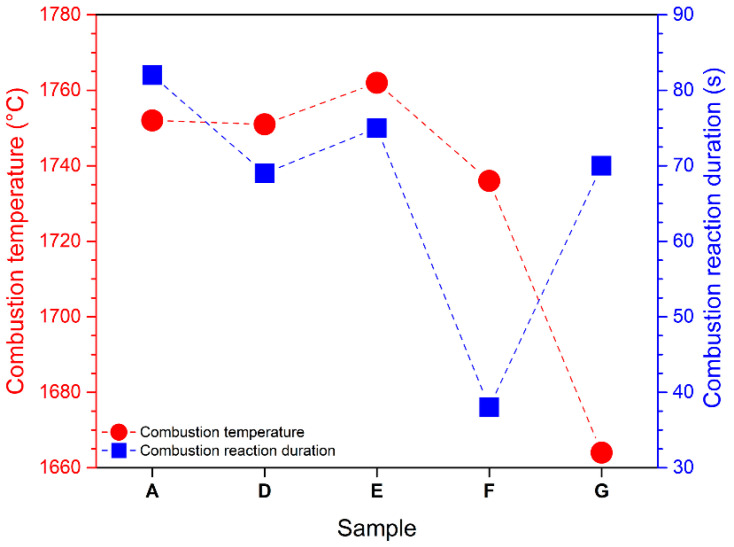
Combustion-reaction temperature and duration.

The temperature–time profile recorded by thermal imaging (Figure 1) indicates that perchlorate-assisted combustion reactions employed for Sr_0.97_Eu_0.01_Dy_0.02_Al_2_O_4_ preparation were highly exothermic. Once the ignition took place (517–541 °C) the temperature increased abruptly, reaching a combustion temperature of 1664–1762 °C, depending on the sample composition (Figure 2).

**Figure 3:**
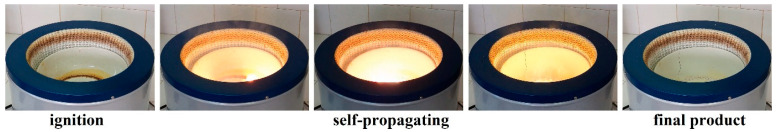
Images captured during the combustion reaction (sample E).

During the combustion process, the sample reached a bright-white incandescence (Figure 3). A large volume of colorless gases evolved during the reaction, leaving behind a white compact solid material.

**Figure 4:**
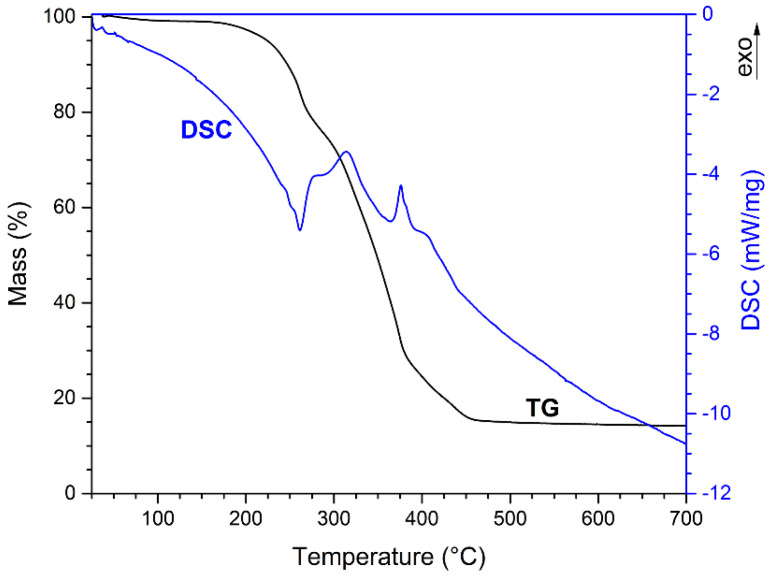
Thermal analysis of precursor mixture F.

Figure 4 shows the TG and DSC curves of precursor mixture F after water evaporation in the drying oven overnight (14 h at 105 °C).

**Figure 5:**
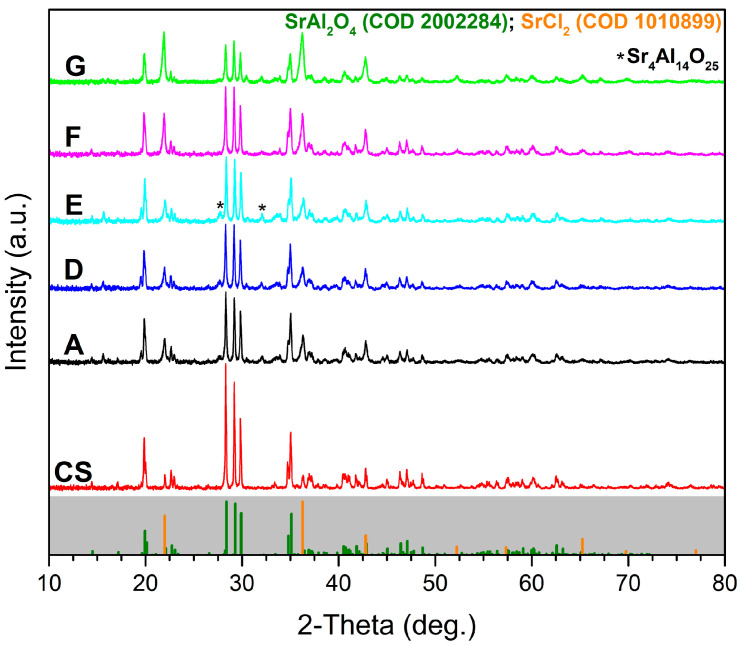
X-ray diffraction patterns of the obtained samples (A, D–G) and commercial sample (CS) samples (color codes: CS—red, A—black, D—blue, E—turquoise, F—magenta, G—green).

The XRD patterns of the samples prepared by combustion synthesis (Figure 5) show the diffraction peaks typical for SrAl_2_O_4_ phase. The XRD pattern of a commercially available SrAl_2_O_4_: Eu^2+^, Dy^3+^ sample (CS) is also shown for comparison.

**Figure 6:**
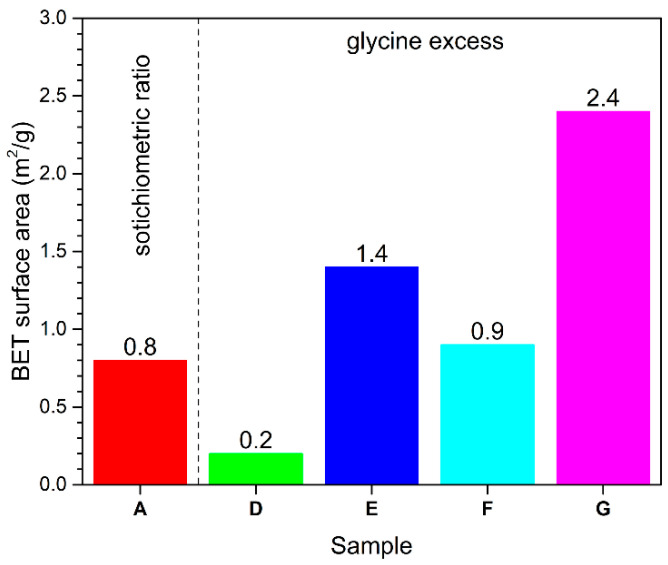
BET surface area of the samples (color codes: A—red, D—green, E—blue, F—turquoise, G—magenta).

Figure 6 shows the BET surface area of combustion-synthesized samples A–G.

The combustion-synthesized samples were characterized and further tested without being subjected to additional annealing under a reducing atmosphere, as frequently mentioned in the literature [9,19,20].

**Figure 7:**
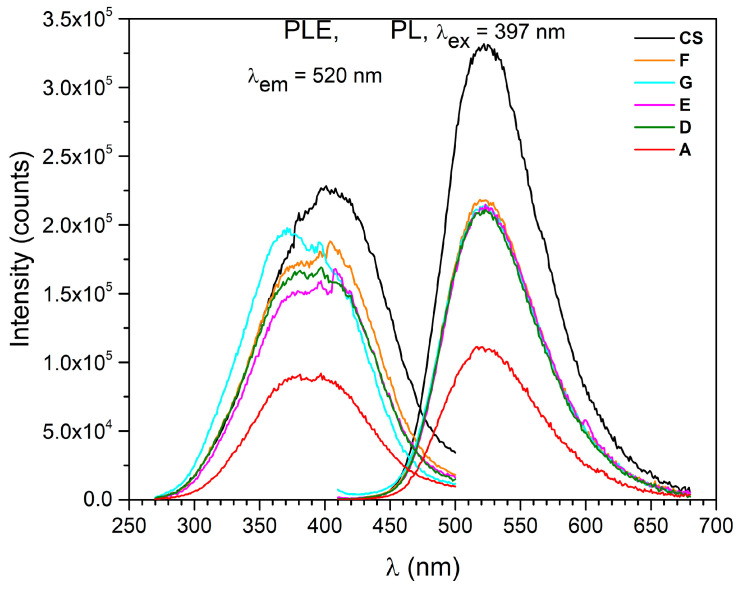
Excitation and emission spectra of the prepared samples by comparison to a commercial sample (CS) samples (color codes: A—red, D—green, E—magenta, F—orange, G—turquoise, CS—black).

The prepared samples were investigated using photoluminescence (PL) and PL kinetics (Figure 7), focusing mostly on how the reducing atmosphere generated by using urea and glycine fuel-rich compositions influenced the optical properties and efficiency of Eu^3+^-to-Eu^2+^ reduction.

**Figure 8:**
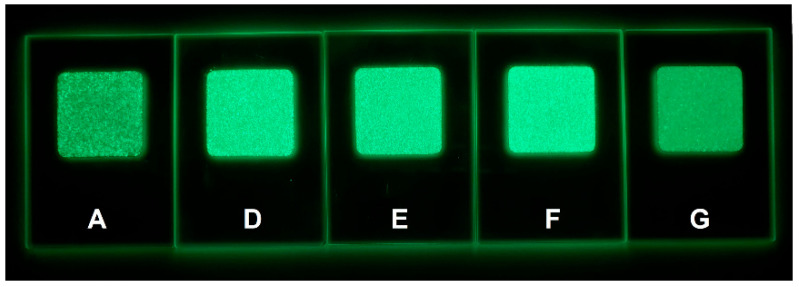
Image in the dark of the prepared samples after irradiation with VIS light.

Images in the dark of the prepared samples after irradiation with VIS light are shown in Figure 8.

**Figure 9:**
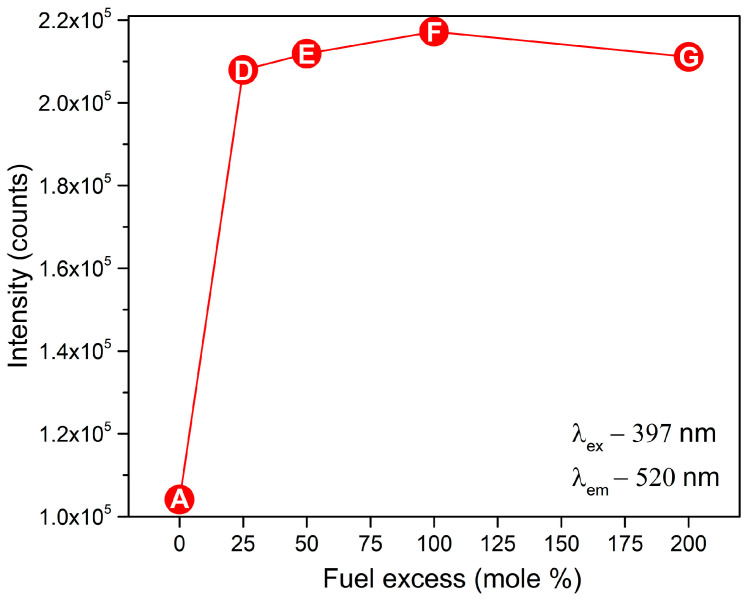
Variation of the PL intensity as a function of fuel excess.

Figure 9 shows a synthetic presentation of the peak emission evolution depending on the glycine-fuel excess (Figure 9).

**Figure 10:**
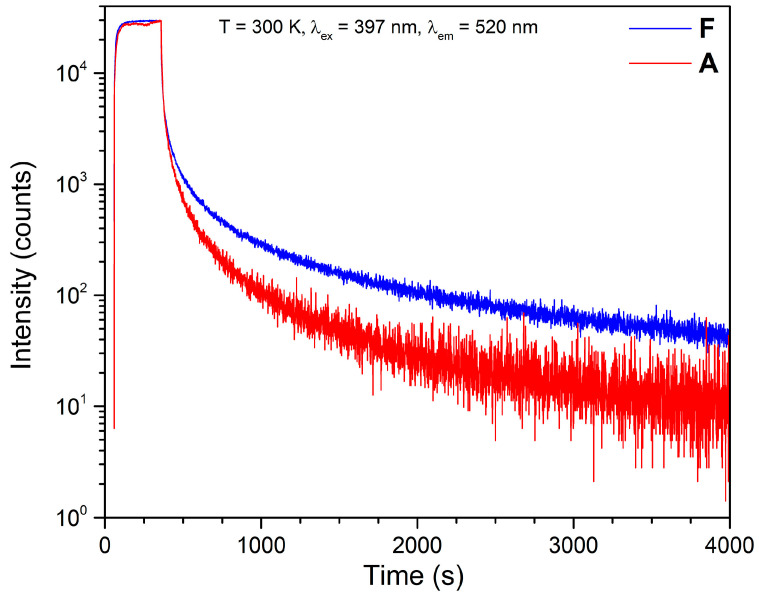
Decay curves of samples A and F.

In order to record the long-lasting phosphorescence and compare the reference sample A and the sample with the highest emission F, the PL-kinetics measurement was performed by pumping samples A and F using a 397 nm wavelength for 5 min followed by switching off the excitation and recording the emission-decay curves for one hour (Figure 10).

The decay curves were analyzed to extract decay-lifetime constants using fitting with double exponential (Equation (1)).

(1) $$I=I_{0}+A1\;\exp\left(\frac{-t}{\tau_{1}}\right)+A2\;\exp\left(\frac{-t}{\tau_{2}}\right)$$

where I_0_ is the initial intensity, t is time, A1 and A2 are the amplitude of the exponents, and τ_1_ and τ_2_ are the decay lifetimes.

The results of the fitting are shown in Table 1. The double exponential fitting gave quite big fitting error to χ^2^ and, to reduce it, we carried out four exponential fittings.

**Figure 11:**
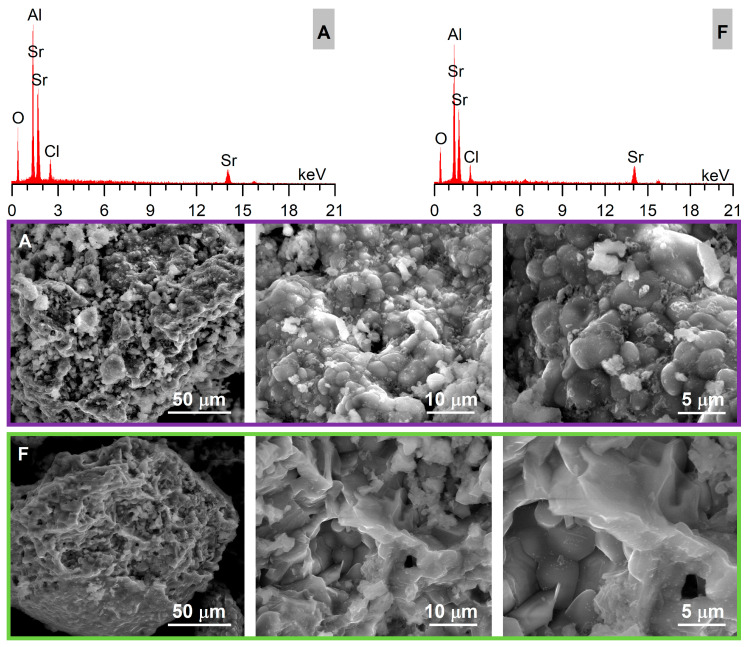
EDX and SEM images of samples A and F.

The EDX and SEM micrographs (Figure 11) of reference sample A (stoichiometric) and sample F (100% glycine excess) are presented for comparison.

**Figure 12:**
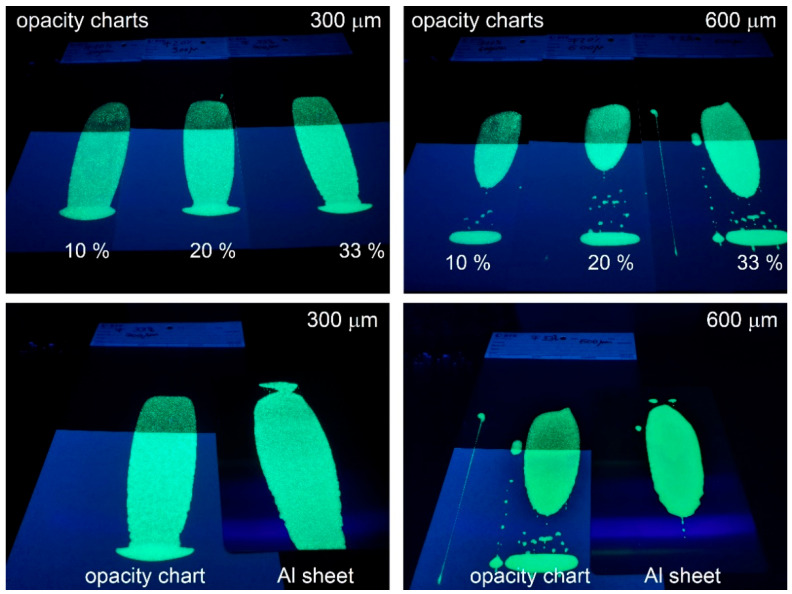
Images of the applied coatings under the 365 nm UV irradiation.

Based on the photoluminescence measurements, sample F, which exhibited the highest emission, was selected for testing in a coating application onto various black/white opacity charts and aluminum sheets. The F pigment was dispersed in an epoxy bi-component transparent resin in different ratios (10, 20, and 33% weight pigment). The prepared coatings were applied in different thickness, using block applicators with 300 and 600 µm gaps. The results are shown in Figure 12.

**Figure 13:**
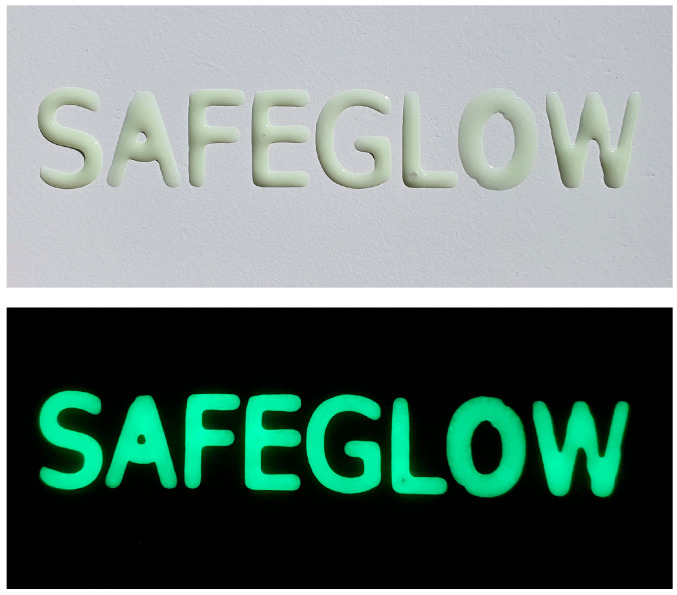
Real-life test of the pigment–organic matrix system prepared with 33% F pigment.

Figure 13 shows the acknowledged project acronym SAFEGLOW in daylight and darkness after illumination with white light for 3 min. The sample was prepared with 33% weight F pigment in the same organic matrix used for the rest of the coatings and it showed very good, lasting visibility in the dark.

## 4. Discussion

The basic stoichiometric combustion reactions, which occur between oxidizers and fuels, can be described by chemical Equations (2)–(5):

9 Sr(ClO_4_)_2_ + 16 C_2_H_5_NO_2_ = 9 SrO + 32 CO_2_ + 31 H_2_O + 18 HCl + 8 N_2_ (2)

18 Eu(NO_3_)_3_ + 32 C_2_H_5_NO_2_ = 18 EuO + 64 CO_2_ + 80 H_2_O + 43 N_2_ (3)

6 Dy(NO_3_)_3_ + 10 C_2_H_5_NO_2_ = 3 Dy_2_O_3_ + 20 CO_2_ + 25 H_2_O + 14 N_2_ (4)

2 Al(ClO_4_)_3_ + 8 CH_4_N_2_O = Al_2_O_3_ + 8 CO_2_ + 13 H_2_O + 6 HCl + 8 N_2_ (5)

As one can observe from the temperature–time profile recorded by thermal imaging, the actual combustion process was extremely fast and took place in a matter of seconds. The combustion-reaction temperature and duration (Figure 2) were slightly influenced by the preparation recipes, e.g., the glycine excess used. The measured temperatures varied within a narrow 100 °C interval for samples A–G, but in terms of reaction velocity, sample E prepared with 100% glycine excess showed the shortest reaction duration (only 38 s). The evolution of the combustion reactions (Figure 3) was characterized by high incandescence and a large volume of evolving gases, and the result was a white, sponge-like reaction product in all cases.

The thermal analysis of precursor-mixture F (Figure 4) revealed an endothermic peak at 261 °C on the DSC curve, accompanied by a mass loss on the TG curve, suggesting the partial decomposition of raw materials. At higher temperature, two exothermic peaks could be seen on the DSC curve at 314 and 376 °C. In both cases, the sample mass decreased, which is consistent with combustion-reaction evolution. Above 450 °C the sample mass reached a constant value and no other exo-/endothermic effects appeared on the DSC curve, confirming that the sample underwent no other transformations. The thermal behavior of other reaction mixtures was very similar.

The main crystalline phase that could be identified based on the XRD analysis (Figure 5) was the monoclinic SrAl_2_O_4_ phase, which was also present in the commercial sample (CS). In comparison to the commercial sample (CS), the obtained samples showed less intense peaks, which suggests the presence of smaller crystallites. The additional peaks that could be seen in the case of the obtained samples indicates the presence of some minor amounts of SrCl_2_. These results are consistent with the energy-dispersive X-ray analysis (Figure 11) showing the presence of Cl in the investigated samples. The presence of SrCl_2_ could be related to the use of perchlorates as oxidizing agents. The alkaline nature of SrO and the formation of HCl as a by-product of the combustion reaction (1) might explain the formation of SrCl_2_. The very small peaks visible on the diffraction patterns at about 27.5° and 32° might be related to the presence of tiny amounts of Sr_4_Al_14_O_25_, which is consistent with other reports in the literature [26]. The phase-composition investigation of the obtained samples (Figure 5) showed that there were no significant differences between the XRD patterns of the five samples; only sample G with the highest content of glycine (200% excess) showed less intense peaks. The sharp profile of the diffraction peaks could be also observed, and it indicated that the high crystallinity of all samples resulted directly from the combustion synthesis without supplementary annealing.

Upon heating, boric acid decomposes into water and boron oxide (6), which basically plays a double role: as a fluxing agent and an optical-properties booster [19,26,27].

2 H_3_BO_3_ = B_2_O_3_ + 3 H_2_O (6)

As a fluxing agent, boron oxide facilitates the formation of the targeted crystalline phase (SrAl_2_O_4_: Eu^2+^, Dy^3+^) by assisting and accelerating solid-state reactions. The second role is related to the potential of B_2_O_3_ to increase the formation of structural defects (e.g., trapping centers), which are highly beneficial for room-temperature long afterglow.

At the same time, the prepared samples exhibited a BET surface area that varied between 0.2 and 2.4 m^2^/g (Figure 6). The samples prepared by perchlorate-assisted combustion synthesis exhibited low BET surface area, which is in agreement with the high temperature measured during the combustion reactions.

The luminescence properties of europium- and dysprosium-doped strontium aluminate are essentially linked to the presence of europium as Eu^2+^, also known as emission centers. The commonly available europium sources, such as europium nitrate–Eu(NO_3_)_3_, contain Eu^3+^. Therefore, measures must be taken to reduce Eu^3+^ to Eu^2+^. Usually, in the case of the ceramic route and other preparation procedures, this reduction process takes place during annealing, which is performed under a reducing atmosphere of hydrogen, active carbon, and carbon monoxide [4,5,6,9,19,20]. However, in the case of combustion synthesis, where no annealing is applied, the reducing atmosphere is artificially created during the combustion reactions by using an over-stoichiometric amount (an excess) of glycine. The excess of glycine consumes the available oxygen inside the furnace, thus enabling a reducing atmosphere. In the case of perchlorate-assisted combustion synthesis, the reduction of Eu^3+^ to Eu^2+^ takes place solely at the expense of the fuel excess, which ensures an active in-situ reducing atmosphere directly during the synthesis process. In addition, the stabilization of Eu^2+^ is facilitated by the elevated combustion temperature and very fast heating and cooling rates (Figure 1).

The samples prepared by the perchlorate-assisted combustion method were very similar to the ones reported in the literature [7,8] for SrAl_2_O_4_: Eu^2+^, Dy^3+^ in terms of excitation and emission spectra. From the peak-position point of view, the excitation and emission spectra of the samples prepared by perchlorate-assisted combustion reaction were also similar to the commercial sample (CS), which is a strong indication for the presence of Eu^2+^ and Dy^3+^. In comparison to the commercial sample, the samples prepared by perchlorate-assisted combustion reaction exhibited a lower emission intensity, which could be related to the lower crystallinity degree confirmed by the XRD analysis (Figure 5). In addition, one also needs to consider that commercial samples are usually heat-treated at elevated temperatures for several hours under a reducing atmosphere, whereas the samples prepared by perchlorate-assisted combustion reaction required no annealing. The recorded PL-excitation curves of the prepared samples consisted of broad bands in the spectral range of 270–500 nm, mostly specific to 4f^6^5d^1^, Eu^2+^ absorption responsible for the emission at 520 nm (Figure 7). Taking into consideration the excitation curves as well as the potential applications of these pigments for glow-in-the-dark safety markings, a 397 nm wavelength was selected for sample pumping. The obtained emission spectra consisted of wide bands with a maximum at 520 nm assigned to Eu^2+^, 4f^6^5d^1^ → 4f^7^ transitions. This is in close agreement with reported data admitting the f-d transition shift to 515 nm due to the crystal-field influence by synthesis or annealing conditions [7,8].

The oxidizer/fuel molar ratio had a very important influence on the emission intensity of the prepared samples. Fuel-rich samples D-G led to a significant increase in the photoluminescence intensity, which was almost doubled in comparison to stoichiometric sample A (Figure 9).

In the case of glycine-rich compositions, the emission intensity gradually increased up to 100% mole of glycine excess (sample F) and then slightly decreased as the glycine excess reached 200% mole (sample G). Among all the prepared samples, sample F showed the highest emission intensity (Figure 9), which was also visible from the image of the prepared samples taken in the dark after irradiation with VIS light (Figure 8). The decrease in the luminescence intensity in the case of sample G may be related to the shift to shorter wavelengths of the Eu^2+^ excitation band (Figure 7). The change in shape of the excitation maximum and UV shifting probably made it less efficient when pumped closer to the VIS range, at 397 nm. The longest decay time was observed in the case of sample F (Figure 10), which also exhibited the highest luminescent intensity. The decay-lifetime constant obtained using four exponential fittings for sample F was τ_avg_ 522.60 s.

The SEM micrographs (Figure 11) of stoichiometric sample A and sample E (100% glycine excess) exhibiting the highest emission showed very similar sponge-like morphology with many voids and agglomerates of sintered coalescent particles, which is additional proof of the high temperature achieved during the combustion reactions (Figure 2). This is a typical morphology for such high-temperature, combustion-synthesized samples and is the result of the high temperatures developed within a short period of time and a large volume of evolved gases. The advanced sintering process observed in the case of samples A and F is also consistent with the low BET surface area of the two samples.

The pigment showed good hiding power in the coatings prepared with 33% wt. (Figure 12). The coatings prepared with lower pigment content also yielded reasonable emissions, but less so, especially on a dark background. As expected, the coatings with 600 µm wet thickness developed better glow-in-the-dark properties on both black/white opacity charts and aluminum sheets. A white or glossy background was noticed to enhance the glowing of the applied coatings due to additional light reflection and pigment excitation.

The real-life test of the pigment–organic matrix system prepared with 33% F pigment (Figure 13) yielded good results after ambient-light illumination of the test sample in terms of both emission intensity and duration, with good visibility in the dark. This confirmed the possibility of using the tested pigment to make glow-in-the-dark safety markings.

## 5. Conclusions

SrAl_2_O_4_: Eu^2+^, Dy^3+^ phosphorescent pigments were prepared by the perchlorate-assisted combustion synthesis, without conducting a subsequent annealing stage.

The influence of oxidizer/fuel molar ratio was investigated using stoichiometric and fuel-rich compositions of urea and glycine-fuel mixtures. During the combustion process, the temperature reached very high values of 1664–1762 °C, depending on the sample composition.

The XRD patterns confirmed the presence of the diffraction peaks typical for SrAl_2_O_4_ in all samples.

The optical properties were highly related to the oxidizer/fuel molar ratio. A clear benefit was noticed in the case of samples prepared with urea- and glycine-fuel-rich recipes.

The active reducing atmosphere created using urea- and glycine-fuel-rich recipes promoted the stabilization of Eu^2+^ and boosted the photoluminescent intensity, which increased by 100% in reference to that in the sample prepared from a stoichiometric mixture.

SrAl_2_O_4_: Eu^2+^, Dy^3+^ phosphorescent pigments were tested in different coatings applied to opacity charts and aluminum sheets. In terms of photoluminescent intensity and decay time, the best results were obtained in the case of the coatings prepared with 33% pigment applied with the 600 µm gap-size block applicator.

## Figures and Tables

**Table 1:** Exponential-fitting results.

Sample	Decay Constants					
	τ_1_ (s)	τ_2_ (s)	τ_3_ (s)	τ_4_ (s)	χ^2^	τ_avg_ (s)
F	46.62	529.67	-	-	3.47	361.40
[28]	46.80	268.79	-	-	-	
[28]	30.25	160.97	-	-	-	
[29]	32.97	62.75	68.22	-	-	68.22
F	9.19	44.77	194.43	972.72	0.73	522.60
[30]	1.29	29.27	221.10	1955.00	1.16	1776.99
[30]	11.58	59.84	243.60	1167.00	1.66	757.25

## Data Availability

Not applicable.
